# Improving Powder Magnetic Core Properties via Application of Thin, Insulating Silica-Nanosheet Layers on Iron Powder Particles

**DOI:** 10.3390/nano7010001

**Published:** 2016-12-23

**Authors:** Toshitaka Ishizaki, Hideyuki Nakano, Shin Tajima, Naoko Takahashi

**Affiliations:** Toyota Central R&D Labs., Inc., 41-1 Nagakute, Aichi 480-1192, Japan; hnakano@mosk.tytlabs.co.jp (H.N.); tajima-angler@mosk.tytlabs.co.jp (S.T.); nao-t@mosk.tytlabs.co.jp (N.T.)

**Keywords:** powder magnetic core, silica nanosheet, insulating layer, electrical resistivity, iron loss, magnetic flux density

## Abstract

A thin, insulating layer with high electrical resistivity is vital to achieving high performance of powder magnetic cores. Using layer-by-layer deposition of silica nanosheets or colloidal silica over insulating layers composed of strontium phosphate and boron oxide, we succeeded in fabricating insulating layers with high electrical resistivity on iron powder particles, which were subsequently used to prepare toroidal cores. The compact density of these cores decreased after coating with colloidal silica due to the substantial increase in the volume, causing the magnetic flux density to deteriorate. Coating with silica nanosheets, on the other hand, resulted in a higher electrical resistivity and a good balance between high magnetic flux density and low iron loss due to the thinner silica layers. Transmission electron microscopy images showed that the thickness of the colloidal silica coating was about 700 nm, while that of the silica nanosheet coating was 30 nm. There was one drawback to using silica nanosheets, namely a deterioration in the core mechanical strength. Nevertheless, the silica nanosheet coating resulted in nanoscale-thick silica layers that are favorable for enhancing the electrical resistivity.

## 1. Introduction

Powder magnetic cores have been extensively studied in recent years due to the many advantages they offer over electromagnetic steel sheets with respect to their isotropic magnetic properties, high electrical resistivity, flexible design, potential for size reduction, and high design flexibility [1]. In order to use powder magnetic cores in AC magnetic field applications, however, it is important to reduce the iron loss, i.e., the sum of eddy current loss and hysteresis loss. Pure iron powders, in particular, are well known to offer a high magnetic flux density at low cost, but with the drawback of low electrical resistance and an associated high eddy current loss [2]. Powder magnetic cores are, therefore, usually fabricated by compacting magnetic powder particles and coating them with insulating layers to prevent an eddy current from forming. This technique of coating powder particles with appropriate insulation is crucial to improving the magnetic properties of a core.

Powder magnetic cores generally need to be annealed after press forming to reduce plastic strain, as this accumulation of strain causes a high hysteresis loss. However, if the insulating layers used do not have sufficient thermal resistance they may break after annealing, resulting in a decrease in electrical resistivity and a very high eddy current loss. Although an increase in the thickness of the insulating layers would enhance their thermal resistance, this would also reduce the magnetic flux density due to the increased volume of non-magnetic phase. Consequently, the powder particles need to be coated with insulating layers that have high thermal and electrical resistance, and which are also thin and homogeneous.

Several insulating materials have been used to coat powder particles, including silicone resins [3], phosphates [4,5,6,7], silicate glasses [8,9], magnesium oxide [10], aluminum oxide [11,12,13], ferrites [14,15,16], sodium salts [17,18], and silica [19,20,21,22,23]. A homogeneous and thin layer can be realized by using silicone resin or phosphate, because these materials undergo chemical reactions with the particle surface. However, the thermal resistance of these materials is not very high, and so they are prone to breakage during annealing. An insulating layer composed of silicate glass offers a high thermal resistance, but the thermal stress generated by the difference in the coefficients of thermal expansion between it and iron causes the magnetic properties to deteriorate. Magnesium and aluminum oxides also have high thermal resistance, but as the deposition of thin, homogenous layers is challenging, the magnetic flux density generally deteriorates due to the extra volume of non-magnetic layers. Ferrites have the advantage of being magnetic and insulating, but their nano-scale deposition with high coverage is still difficult to achieve by ordinary methods involving the mixing of ferrite particles or chemical precipitation. An insulating layer composed of sodium salts has a high thermal resistance and can be formed homogeneously, but pure iron is prone to corrosion by sodium salts. Of the various insulating materials used for magnetic materials, silica provides excellent stability against corrosion, oxidation, and a high thermal-resistance [22,23,24,25,26]. Silica coating of magnetic powder particles is often carried out via the hydrolyzation of tetraethoxysilane (TEOS), but this method makes it difficult to achieve thin, homogeneous layers of silica [19,20,21,22,23]. An appropriate technique for creating the homogenous, nanoscale silica layers needed for magnetic powder cores has, to the best of our knowledge, not yet been reported.

Tajima et al. developed a new type of insulating layer composed of strontium phosphate and boron, and showed that this has a higher electrical resistivity than conventional insulating layers composed of phosphates, even after annealing at 673 K (this layer is hereafter referred to as a Sr-B-P-O insulating layer) [27]. However, the thermal resistance of this Sr-B-P-O insulating layer was still insufficient to remove strain by annealing at high temperature, and so an attempt was made to enhance its thermal resistance by mixing silica compounds into iron powders coated with Sr-B-P-O insulating layers. This resulted in a high electrical resistivity, but at the cost of a substantial decrease in the compact and magnetic flux densities. The thickness of the silica coating, therefore, needs to be reduced, and its homogeneity increased, by adopting an effective technique for precise deposition on iron surfaces. There have, however, been very few studies in which the deposition of thin insulating layers of silica have been deposited on iron powder particles for fabricating a magnetic core, as there has been no effective method of achieving thin silica layers with precise control over their deposition.

A new inorganic-layer coating technique that involves the alternating deposition of cationic polymers and negatively-charged colloids on a solid surface has attracted much attention of late because it allows the layer thickness to be controlled by simply changing the number of coatings. This coating technique is based on the layer-by-layer (lbl) assembly of oppositely-charged polyelectrolyte and colloidal inorganic nanoparticles to create a multilayer film [28,29]. Using this technique, oxide nanosheets were coated onto inorganic substrates in a colloidal suspension, from which a variety of unique optical and electronic properties were obtained by precisely controlling the coating thickness [30,31,32,33]. It can, therefore, be hypothesized that thin insulating layers with high thermal and electrical resistivities could be realized through the precise deposition of nanoscale silica onto magnetic powder particles.

Although commercial suspensions of colloidal silica are currently available, their particle diameter is normally more than 10 nm. Thus, in order to reduce the size of silicon-based materials, Nakano et al. synthesized nanosheets with a nanometer scale thickness composed of silicon and amorphous silica by exfoliating layered polysilane to create colloidal monolayers [34,35,36]. If these very thin silica nanosheets could be coated onto magnetic powder particles, they should provide an effective insulating layer. To test this idea, silica was coated onto iron powder particles via the lbl method using poly(diallyldimethylammonium chloride) (PDADMAC) as the cationic polymer, and the magnetic properties of the magnetic cores produced from this material were examined.

## 2. Results and Discussion

### 2.1. Colloidal Silica Coating of Iron Powder Particles with and without Sr-B-P-O Insulating Layers

In order to assess the adhesiveness of silica, pure iron powder particles with a diameter of 20–160 μm were used with or without Sr-B-P-O insulating layers, which was produced using a previously described method [27]. The colloidal silica with a diameter of 10–20 nm was coated onto pure iron powder particles with or without Sr-B-P-O insulating layers after coating of PDADMAC, and this process was repeated 1–5 times. Toroidal cores with outer and inner diameters of 39 and 30 mm and a thickness of 5 mm were fabricated by warm compaction of the coated iron powders at 423 K with a pressure of 1176 MPa using the die wall lubrication method [37,38]. These toroidal cores were then annealed for 30 min at 773 K in a nitrogen atmosphere to remove plastic strain.

**Figure 1:**
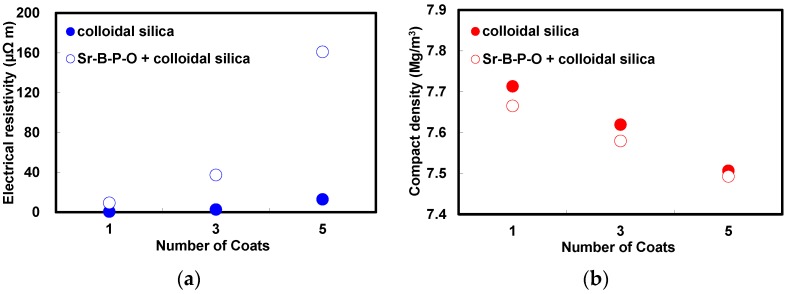
(**a**) Electrical resistivities; and (**b**) compact densities of the annealed toroidal cores fabricated from iron powder particles coated with colloidal silica with and without Sr-B-P-O insulating layers increasing number of silica coatings from 1–5.

Figure 1 provides the comparison of the electrical resistivities and compact densities of the annealed toroidal cores fabricated from iron powder particles coated with colloidal silica with and without Sr-B-P-O insulating layers increasing number of silica coatings from 1 to 5. Note that when the iron powder particles were directly coated with colloidal silica, the electrical resistivity did not increase appreciably with an increasing number of silica coatings, but the compact density decreased proportionally. When Sr-B-P-O insulating layers were applied prior to coating with colloidal silica the electrical resistivity increased by a much greater extent, though the decrease in compact density was much the same in both cases. This indicates that amount of colloidal silica deposited was almost the same regardless of whether Sr-B-P-O insulating layers were used or not, but those particles with Sr-B-P-O insulating layers could be coated with silica more effectively. A higher resistivity could, therefore, be obtained by coating silica over Sr-B-P-O insulating layers, and after every cycle of coating, the compact density was found to be slightly lower than the particles without Sr-B-P-O insulating layers. We can conclude from this that Sr-B-P-O insulating layers can accelerate the adsorption of colloidal silica, which should result in a core with a higher electrical resistivity and lower compact density.

The obtained toroidal cores were used for evaluation of the magnetic properties because they can prevent the influence of a demagnetizing field. The magnetic properties were measured by DC and AC B-H curve tracers. Magnetic flux densities and maximum permeabilities were estimated at a magnetic field strength of 10 kA/m from the DC B-H curves. Coercivities were also estimated from the DC B-H curves applying a magnetic field strength to be 2 kA/m. Hysteresis and eddy current losses were estimated at the maximum magnetic flux density of 1 T and frequency of 400 Hz by an AC B-H curve tracer. An iron loss was the sum of hysteresis and eddy current losses.

**Figure 2:**
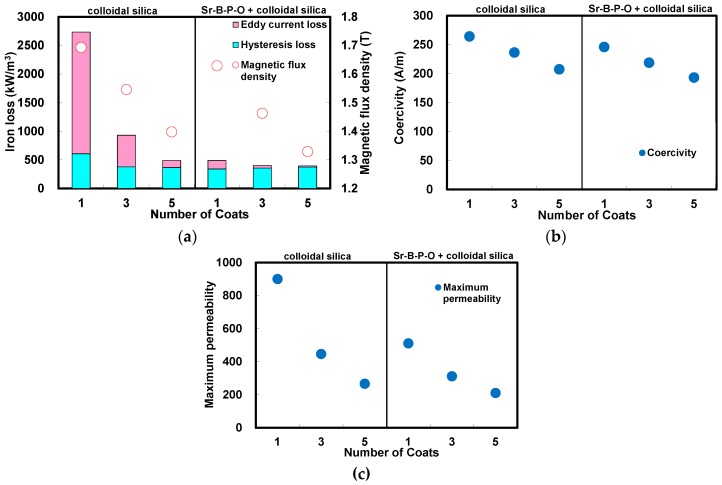
(**a**) Iron losses, magnetic flux densities; (**b**) coercivities; and (**c**) maximum permeabilities of the annealed toroidal cores fabricated from iron powder particles coated with colloidal silica with and without Sr-B-P-O insulating layers increasing number of silica coatings from 1 to 5.

Figure 2 shows the comparison of the iron losses, magnetic flux densities, coercivities and maximum permeabilities of the annealed toroidal cores fabricated from iron powder particles coated with colloidal silica with and without Sr-B-P-O insulating layers increasing number of silica coatings from 1 to 5. The magnetic hysteresis curves measured by a DC B-H curve tracer are shown in Figures S1 and S2 for the particles with and without Sr-B-P-O insulating layers after coating of colloidal silica, respectively. The decreasing trend in magnetic flux density with increasing number of silica coatings was almost the same whether Sr-B-P-O insulating layers were used or not, but as both coatings are non-magnetic, there was greater degradation in magnetic flux density when Sr-B-P-O insulating layers were used in addition to silica. This agrees well with the decreasing trend in the compact densities, which is closely related to the magnetic flux density. The eddy current losses also decreased with increasing number of silica coatings, regardless of whether Sr-B-P-O insulating layers were used or not, and the hysteresis losses were also almost the same. However, when the particles with Sr-B-P-O insulating layers were used, the eddy current loss after every coating cycle was substantially less than with the particles without Sr-B-P-O insulating layers. This can be explained by the fact that the eddy current loss decreases in inverse proportion to the electrical resistivity, which was higher when silica was coated over Sr-B-P-O insulating layers. The coercivities of the cores decreased with increasing number of silica coatings, as did the compact and magnetic flux densities. This suggests that silica layers provide a barrier against plastic strain during fabrication, and as coercivity is dependent on plastic strain, it is gradually reduced with an increase in the thickness of the silica coating applied. The permeabilities of the cores also decreased with the increasing number of silica coatings, because a permeability is generally reduced owing to increase of an amount of silica.

**Figure 3:**
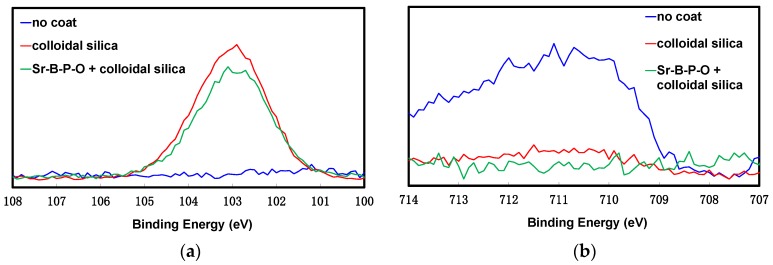
X-ray photoelectron spectroscopy (XPS) (**a**) Si2p; and (**b**) Fe2p3/2 core level spectra of uncoated iron powder particles and iron power particles coated five times with colloidal silica with and without Sr-B-P-O insulating layers.

The above results suggest that irrespective of the surface condition, the similar amount of silica is deposited onto surfaces with each increase in the number of silica coatings. XPS (X-ray photoelectron spectroscopy) analysis was conducted on uncoated iron powder particles and iron powder particles coated five times by colloidal silica with and without Sr-B-P-O insulating layers in order to analyze the surface chemical states. The Si2p and Fe2p3/2 core level spectra in Figure 3 show a strong peak corresponding to silica (SiO_2_) at around 103 eV for the particles with and without Sr-B-P-O after coating with colloidal silica, which indicates that silica can be deposited regardless of whether an insulating layer exists or not. A strong peak corresponding to iron oxides (Fe_2_O_3_, Fe_3_O_4_) was observed at around 710–712 eV in the case of the uncoated particles, and this is attributed to naturally-formed surface oxides. A small peak corresponding to iron oxides was also observed with the particles coated only with colloidal silica, but this was weaker than that of the uncoated particles. This peak corresponding to iron oxides was barely visible for the particles coated with colloidal silica over Sr-B-P-O insulating layers. As the analysis depth of XPS is only about 2 or 3 nm, iron oxides cannot be detected if the surface is thoroughly coated with colloidal silica. This means that the particles without Sr-B-P-O insulating layers were not completely covered by silica, as the uncoated surface was detected by XPS, whereas no surface was exposed on particles with Sr-B-P-O insulating layers. This difference agrees well with the experimental results for the electrical resistivities and eddy current losses between the cores prepared from particles coated with colloidal silica with and without Sr-B-P-O insulating layers.

**Figure 4:**
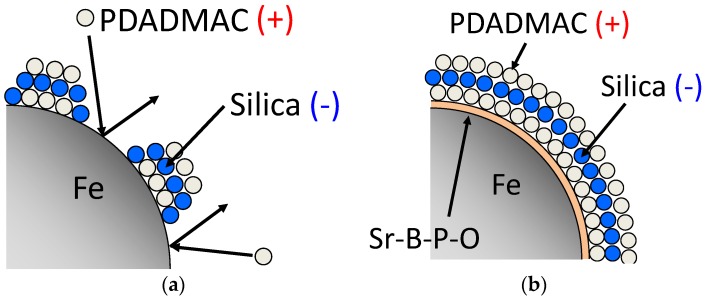
Schematic models for the silica coating of iron powder particles by the lbl method (**a**) without; and (**b**) with Sr-B-P-O insulating layers.

Schematic models for coating powder particles via the lbl method, with and without Sr-B-P-O insulating layers, are shown in Figure 4. Note that when no Sr-B-P-O insulating layers are used, PDADMAC is not adequately attracted to the overall surface during the first coating step. However, those areas that are covered by PDADMAC succeed in attracting silica, and become repeatedly coated with silica with each coating cycle. This coating process results in islands of insulating layers, which do not increase the electrical resistivity, even though the density does decrease. Powder particles with Sr-B-P-O insulating layers, on the other hand, attract much more PDADMAC during the first step, with silica being subsequently deposited on these PDADMAC-covered surfaces to produce robust insulating layers.

### 2.2. Comparison between Coloidal Silica and Silica Nanosheet Coatings

Although coating with Sr-B-P-O was very effective in increasing the electrical resistivity of the toroidal core prepared with silica-coated iron particles, the compact and magnetic flux densities deteriorated considerably with increasing number of colloidal silica coatings. The colloidal silica particles used in the experiment had an average diameter of 10–20 nm, and so the resulting silica layers were simply too thick to achieve a high compact density with increasing coating cycles. Ideally, any increase in the electrical resistivity should not reduce the high compact and magnetic flux densities. This can be made possible by using silica nanosheets to coat the iron powder particles. Silica nanosheets were synthesized by the same procedure described in a previous paper [35]. The detailed characterization of silica nanosheets was also shown in the same paper [35]. The thickness per sheet was 0.68 nm and the length was in the range of 100–200 nm, which were observed by TEM (transmission electron microscopy) and AFM (atomic force microscopy). The selected-area electron diffraction indicated that the crystal structure was amorphous state.

**Figure 5:**
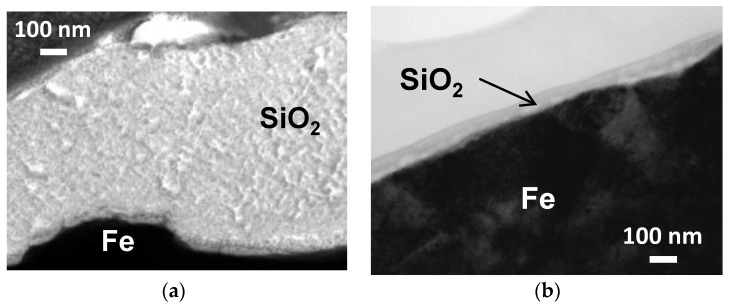
TEM images of silica layers on iron powder particles with Sr-B-P-O insulating layers coated five times by (**a**) colloidal silica; and (**b**) silica nanosheets.

Figure 5 shows cross-sectional TEM images of iron powder particles with Sr-B-P-O insulating layers after five coatings of colloidal silica and silica nanosheets. These images reveal that the silica layer thicknesses were about 700 nm in the case of the colloidal silica layer, but just 30 nm for the silica nanosheet layer. This thickness of the silica nanosheet layers was much less than has been achieved via the hydrolyzation of TEOS [20,21], which means that significantly thinner silica layers can be obtained by using silica nanosheets compared with the conventional method. We consider that our process by the lbl method of silica nanosheets over Sr-B-P-O insulating layers has the advantage that deposition of silica can be more precisely controlled, resulting in thinner layers.

**Figure 6:**
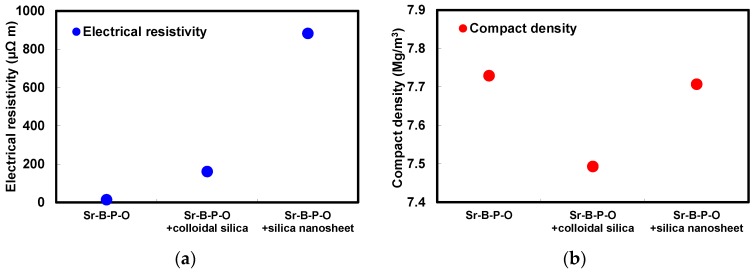
(**a**) Electrical resistivities; and (**b**) compact densities of annealed toroidal cores fabricated from iron powder particles coated only with Sr-B-P-O insulating layers, and with five coatings of colloidal silica or silica nanosheets over Sr-B-P-O insulating layers.

Figure 6 shows the comparison of the electrical resistivities and compact densities of the annealed toroidal cores fabricated from iron powder particles coated only with Sr-B-P-O insulating layers, and those with five coatings of colloidal silica or silica nanosheets over Sr-B-P-O insulating layers. When only Sr-B-P-O insulating layers were used, the electrical resistivity was low, but the compact density was high. The Sr-B-P-O insulating layers were thin, but could break after annealing because of their insufficient thermal resistance, resulting in a low electrical resistivity. However, the electrical resistivity increased and the compact density decreased significantly after a coating of colloidal silica was applied over the Sr-B-P-O insulating layers. This indicates that the insulative properties were reinforced by colloidal silica, resulting in a higher electrical resistivity, but the extra volume of silica reduced the compact density because the silica layers were too thick. When silica nanosheets were coated over Sr-B-P-O insulating layers instead, the electrical resistivity increased remarkably, while the compact density decreased very little compared to the particles with only Sr-B-P-O insulating layers. This means that not only were the silica nanosheet layers thin enough to maintain a sufficiently high compact density, they also had a higher thermal resistivity than the Sr-B-P-O insulating layers and colloidal silica over Sr-B-P-O insulating layers. In fact, it is worth noting that the electrical resistivity was more than 800 μΩm, even after annealing.

**Figure 7:**
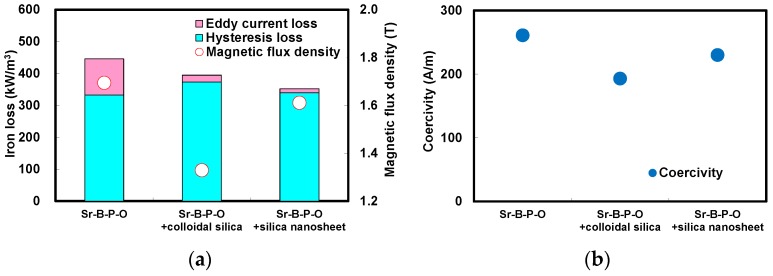

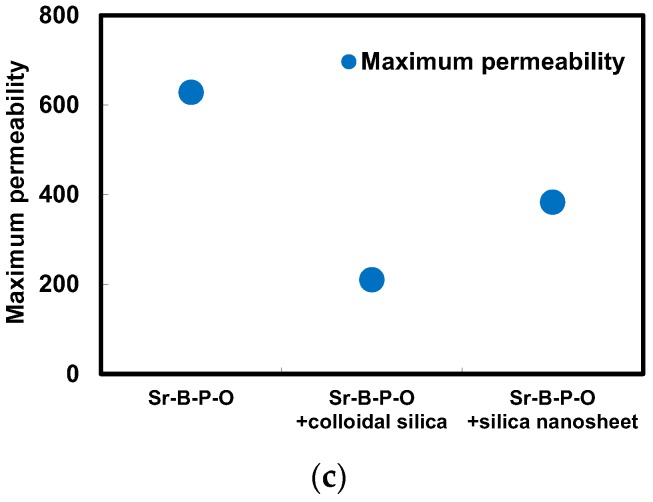
(**a**) Iron losses, magnetic flux densities; (**b**) coercivities; and (**c**) maximum permeabilities of annealed toroidal cores fabricated from iron powder particles coated only with Sr-B-P-O insulating layers, and with five coatings of colloidal silica or silica nanosheets over Sr-B-P-O insulating layers.

Figure 7 presents the comparison of the iron losses, magnetic flux densities, coercivities, and maximum permeabilities of the annealed toroidal cores fabricated from iron powder particles coated only with Sr-B-P-O insulating layers, and with five coatings of colloidal silica or silica nanosheets over Sr-B-P-O insulating layers. The magnetic hysteresis curves measured by a DC B-H curve tracer are shown in Figure S3. In the case of powder particles with only Sr-B-P-O insulating layers, the magnetic flux density was high despite a high iron loss caused by the high eddy current loss. This eddy current loss was reduced by coating colloidal silica over Sr-B-P-O insulating layers, but the magnetic flux density also decreased considerably. However, the coercivity also decreased with colloidal silica coating, because the thick silica layers could prevent the accumulation of plastic strain. The silica nanosheet coating, on the other hand, produced the lowest iron loss. The hysteresis loss was the similar magnitude between applying only Sr-B-P-O insulating layers (332 kW/m^3^), colloidal silica (373 kW/m^3^) and silica nanosheets over Sr-B-P-O insulating layers (340 kW/m^3^). However, the eddy current loss with silica nanosheets was significantly low (12 kW/m^3^) than with only Sr-B-P-O insulating layers (114 kW/m^3^) and colloidal silica (21 kW/m^3^). Since an eddy current loss is dependent on a resistivity, the eddy current loss became the lowest value after coating with silica nanosheets. It is significant for the magnetic core that the iron loss could be reduced about 20% by coating silica nanosheets over Sr-B-P-O insulating layers, resulting in improving the performance. The magnetic flux density with silica nanosheet coating over Sr-B-P-O insulating layers was also high due to the low compact density. These magnetic losses and flux densities agree well with the electrical resistivities and compact densities for the three powders. The fact that the coercivity was more greatly reduced with silica nanosheet coating over Sr-B-P-O insulating layers compared to using only Sr-B-P-O insulating layers indicates that that the silica nanosheet layers also prevented the accumulation of plastic strain. However, the coercivity after coating with silica nanosheets was slightly higher than when colloidal silica was used, because the greater thickness of the colloidal silica layers allowed more plastic strain to be removed. The result about the permeabilities also indicates the same trend as did the compact and magnetic flux densities, because a permeability is dependent on the composition ratio of iron.

**Figure 8:**
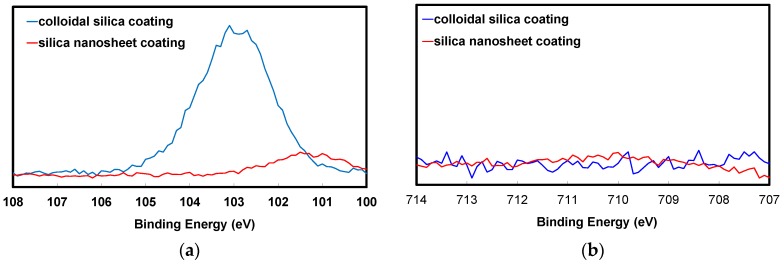
XPS (**a**) Si2p; and (**b**) Fe2p3/2 core level spectra of iron powder particles coated five times with colloidal silica or silica nanosheets over Sr-B-P-O insulating layers.

Figure 8 shows the Si2p and Fe2p3/2 core level spectra of iron powder particles that were coated five times with colloidal silica or silica nanosheets over Sr-B-P-O insulating layers. The strong peak that was observed at around 103 eV after coating with colloidal silica confirms that SiO_2_ layers were formed on the powder surface [39]. In contrast, a weak peak at around 101 eV was observed after coating with silica nanosheets. This peak obviously represents a shift in the SiO_2_ peak to a lower binding energy, indicating that the Si oxidation state in the silica nanosheet layer was lower than that of SiO_2_. A previous paper reported that silica nanosheets were amorphous and had an atomic ratio of Si:O of about 1:1.8 [35]. This is consistent with another study that reported that the peak for amorphous silica was observed at a lower-energy than that of SiO_2_ [39]. As amorphous silica is also reported to be a superior insulator, we believe that the electrical resistivity can be enhanced more significantly with a silica nanosheet coating than with a colloidal silica coating [40]. The Fe2p3/2 core level spectra did not show any peaks in the case of the particles coated by colloidal silica and silica nanosheets over Sr-B-P-O insulating layers, suggesting that both produced a thorough surface coating that greatly reduced the eddy current loss.

**Figure 9:**
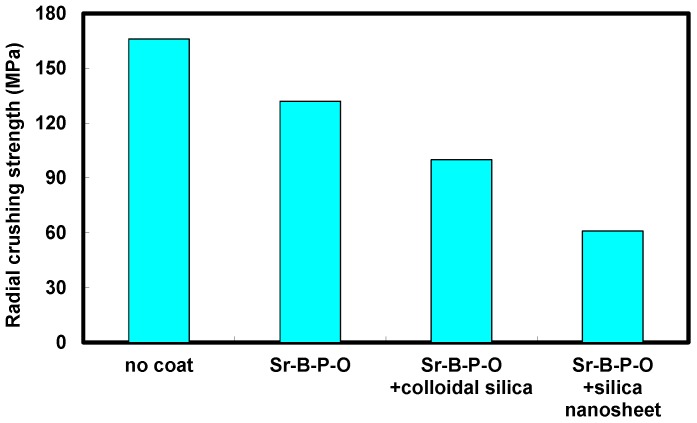
Radial crushing strengths of annealed toroidal cores fabricated from uncoated iron powder particles, particles with only Sr-B-P-O insulating layers, and particles with five coatings of colloidal silica or silica nanosheets over Sr-B-P-O insulating layers.

The mechanical strength of annealed toroidal cores was measured by radial crushing tests. As shown in Figure 9, the highest strength was achieved with the core prepared using uncoated powder particles, with Sr-B-P-O coating and subsequent silica coating both reducing the strength. The toroidal core fabricated using particles coated with silica nanosheets exhibited the lowest strength of less than 60 GPa. The reason for this reduction in strength when using silica nanosheets is not yet understood, and so will be studied in the future.

The above results prove that toroidal cores made from iron powder particles with Sr-B-P-O insulating layers and a silica nanosheet coating provide a good balance between low iron loss and high magnetic flux density. These excellent properties are attributed to the very thin, homogeneous and highly thermal resistant layer that is obtained by coating more precisely than is possible with other methods. The low thickness results in a high compact density, while the homogeneity of the coating results in a high electrical resistivity. Notably, coating with silica nanosheets can overcome the usual trade-off problem of an increasing thickness of insulating layers creating a higher electrical resistivity and lower magnetic loss, but the extra volume of insulating layers reducing the compact density and magnetic flux density.

## 3. Materials and Methods

### 3.1. Preparation of Polycation and Silica Solutions

A 20 wt % solution of PDADMAC (molecular weight 200,000–350,000) produced by Sigma-Aldrich (St. Louis, MO, USA) was used as the polycation solution. The concentration of this PDADMAC solution was adjusted to 0.1 wt % by mixing it with distilled water, and the pH was adjusted to 11 by adding 1 N KOH to prevent the severe corrosion of iron that occurs in solutions with a pH < 11.

A 20 wt % suspension of colloidal silica produced by Nissan Chemical Industries (Chiyoda, Tokyo, Japan) was used for the coating experiment. The average diameter of these colloidal silica particles was about 10–20 nm. The concentration of the silica solution was adjusted to 2 wt % by mixing it with distilled water, and the pH was adjusted to 11 by the addition of 1 N KOH solution.

A suspension containing silica nanosheets was synthesized using a previously described procedure [35], whereby 1 g of CaSi_2_ crystallites produced by Sigma-Aldrich (St. Louis, MO, USA) was first immersed in 100 mL of 37% HCl at 243 K. After stirring continuously in a dark room for seven days under an Ar atmosphere, the mixture was washed with concentrated HCl at 243 K, rinsed with acetone, and then dried at 300 K to synthesize laminated polysilane (SiH)*_n_*. The chemical reaction for this production of laminated polysilane is as follows:

3CaSi_2_ + 6HCl → Si_6_H_6_ + 3CaCl_2_

About 0.5 g of polysilane was mixed with 100 mL of sodium dodecylsulfate (SDS, C_12_H_25_OSO_3_Na) solution produced by Sigma-Aldrich (St. Louis, MO, USA). After shaking this solution at 100 rpm and 300 K for 7 days, a suspension of silica nanosheets was obtained. This suspension was washed with distilled water and centrifuged at 4000 rpm for 10 min in order to remove the SDS and HCl. The concentration of the silica nanosheets was then adjusted to 0.2 wt % by mixing with distilled water, and the pH was adjusted to 11 by the addition of 1 N KOH solution.

### 3.2. Coating Process

Water-atomized powders of pure iron (Fe 99.9 wt %) with an average particle diameter of 20–160 μm produced by JFE Steel (Chiyoda, Tokyo, Japan) were employed to fabricate the powder magnetic cores. Plain powders and powders with Sr-B-P-O insulating layers were prepared in order to compare the adhesiveness of silica. The Sr-B-P-O coating was carried out using the same method described in another paper [27]. The ratio of Sr-B-P-O insulating layers to iron powders was 2 wt %.

The powders were coated with silica layers by first mixing 45 g of the iron powder and 50 mL of PDADMAC solution in a glass bottle by shaking using a rotor at 60 rpm for 20 min. After removing the PDADMAC solution, the powder was mixed with 50 mL of colloidal silica suspension or silica nanosheet suspension and shaken by the same process. The powders were then rinsed using distilled water and collected after removing the distilled water by decantation. This coating process was repeated 1–5 times to prepare multilayer coatings, after which the powders were dried at 373 K under vacuum.

### 3.3. Preparation of Powder Magnetic Cores and Evaluation of Their Magnetic Properties and Mechanical Strength

Powder magnetic cores were prepared via warm compaction by the die wall lubrication method [37,38]. The heating temperature of the die and powder was 423 K. The compacting pressure was 1176 MPa. Every specimen was fabricated into a ring shape to create a toroidal core and prevent the influence of a demagnetizing field; the outer and inner diameters of this core were 39 mm and 30 mm, and the thickness was 5 mm. Every specimen was annealed for 30 min at 773 K in a nitrogen atmosphere to remove plastic strain. The compact density after annealing was measured by Archimedes’ method, and the electrical resistivity was measured with a micro-ohmmeter using a four-point method. Cu wires with a diameter of 0.5 mm were wounded around the toroidal core. The numbers of wire turns were 180 for excitation and 90 for detection, respectively. The magnetic properties of the toroidal core were measured by using DC and AC B-H curve tracers with a TRF-5A-PC (Toei Industry, Machida, Tokyo, Japan) and SY8232 (Iwatsu Test Instruments, Suginami, Tokyo, Japan). Magnetic flux densities and maximum permeabilities were measured under a magnetic field of 10 kA/m using the DC B-H curve. Coercivities were also estimated from the DC B-H curves applying a magnetic field strength to be 2 kA/m. Hysteresis and eddy current losses were measured at the maximum magnetic flux density of 1 T and a frequency of 400 Hz using the AC B-H curve tracer. It is known that an iron loss (*P_c_*) can be calculated by the sum of a hysteresis loss (*P_h_*) and an eddy current loss (*P_e_*) with a frequency (*f*) as a parameter by the following Equation (1):

(1) $$P_{c}=P_{h}\times f+P_{e}\times f^{2}$$

Hysteresis and eddy current losses were separately estimated from frequency dependency of iron loss by the Equation (1). The radial crushing strength of the toroidal core was measured using the press mode of an electronic universal testing machine (Shimadzu, Kyoto, Kyoto, Japan).

### 3.4. Observation and Surface Analyses

TEM observation with a JEM 2010FEF (JEOL, Akishima, Tokyo, Japan) was conducted to observe the thickness of the silica layers formed using colloidal silica and silica nanosheets. Samples for this TEM observation were cross-sectionally polished using the FIB (focused ion beam) method with a FB-2000A (Hitachi High-Technologies, Minato, Tokyo, Japan). XPS analysis was conducted to investigate the chemical state of the coating layers and compare the surface conditions of the uncoated powder particles, powder particles directly coated with colloidal silica, and powder particles with Sr-B-P-O and colloidal silica or silica nanosheet coatings. The analysis region and depth were approximately φ100 μm and 2–3 nm, respectively. The XPS instrument used was a Quantera SXM (ULVAC-PHI, Chigasaki, Kanagawa, Japan) with a monochromatic Al Kα source. Charge-up compensation was made using C1s (285 eV).

## 4. Conclusions

When iron powder particles were directly coated by colloidal silica, the compact density of the toroidal core fabricated from them decreased due to the silica deposition, but the electrical resistivity could not be increased because the silica layers existed as islands over the iron surface and did not provide complete insulation. In contrast, Sr-B-P-O insulating layers on iron particles were more capable of attracting silica and became completely coated, resulting in an effective insulation layer. The magnetic core fabricated from iron powder particles with Sr-B-P-O insulating layers and colloidal silica coating exhibited low iron loss due to the high electrical resistivity, but the magnetic flux density decreased considerably because of the excessively thick silica layers (about 700 nm). When silica nanosheets were used as the coating material instead of colloidal silica, the silica layer thickness was reduced to only 30 nm. This thickness was much less than has been achieved by the conventional method. A lower iron loss could therefore be achieved due to the higher electrical resistivity and higher magnetic flux density made possible by the thinner silica layers. The drawback to this approach was that the strength of the core was reduced after silica nanosheet coating. Nevertheless, it can be concluded that silica nanosheet coating results in amorphous, nanoscale-thickness silica layers that are favorable for enhancing the electrical resistivity of iron powder magnetic cores.

## Supplementary Materials

The following are available online at http://www.mdpi.com/2079-4991/7/1/1/s1, Figure S1: Magnetic hysteresis curves of the annealed toroidal cores fabricated from uncoated iron powder particles after coating one, three, and five times with colloidal silica, Figure S2: Magnetic hysteresis curves of the annealed toroidal cores fabricated from iron powder particles with Sr-B-P-O insulating layers after coating with one, three, and five times with colloidal silica, Figure S3: Magnetic hysteresis curves of annealed toroidal cores fabricated from iron powder particles with only Sr-B-P-O insulating layers, and those with colloidal silica and silica nanosheets coated five times over the Sr-B-P-O insulating layers.
