# Supplementary material for: Improving Powder Magnetic Core Properties via Application of Thin, Insulating Silica-Nanosheet Layers on Iron Powder Particles
Source: Nanomaterials (Basel). 2016 Dec 23;7(1):1. doi: 10.3390/nano7010001 (PMC5295191; doi:10.3390/nano7010001)
Supplement: Supplementary file 1 [file nanomaterials-07-00001-s001.pdf]

Supplementary Materials: Improving Powder Magnetic Core Properties via Application of Thin, Insulating Silica-Nanosheet Layers on Iron Powder Particles

Toshitaka Ishizaki, Hideyuki Nakano, Shin Tajima and Naoko Takahashi

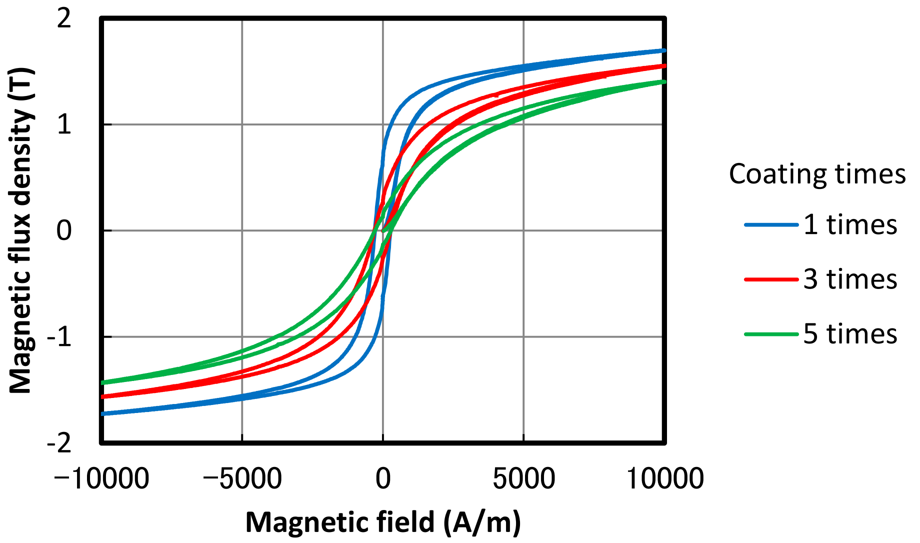

Figure S1. Magnetic hysteresis curves of the annealed toroidal cores fabricated from uncoated iron powder particles after coating one, three, and five times with colloidal silica.

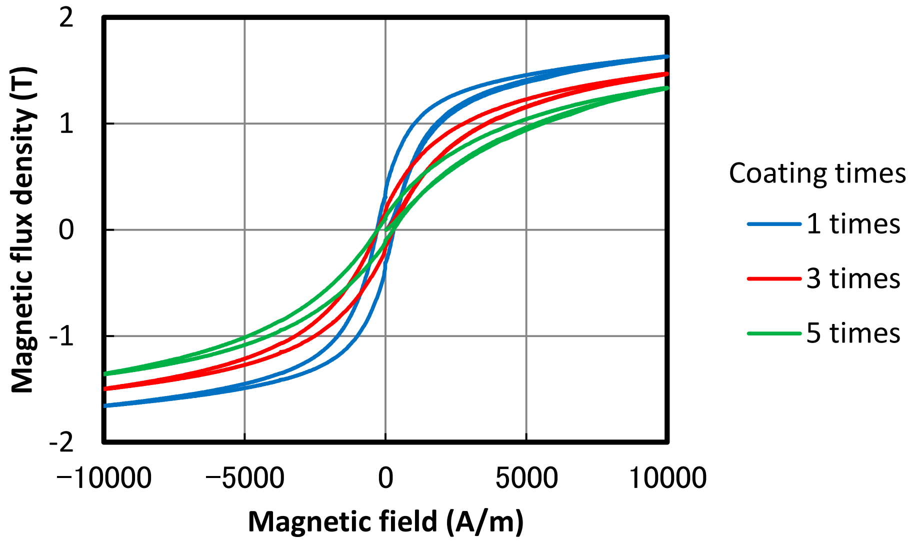

Figure S2. Magnetic hysteresis curves of the annealed toroidal cores fabricated from iron powder particles with Sr-B-P-O insulating layers after coating with one, three, and five times with colloidal silica.

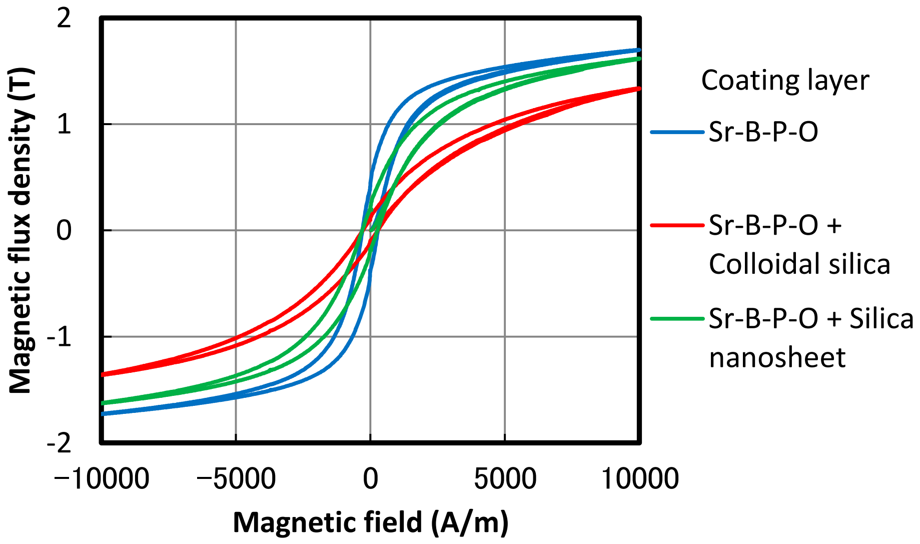

Figure S3. Magnetic hysteresis curves of annealed toroidal cores fabricated from iron powder particles with only Sr-B-P-O insulating layers, and those with colloidal silica and silica nanosheets coated five times over the Sr-B-P-O insulating layers.

© 2016 by the authors. Submitted for possible open access publication under the terms and conditions of the Creative Commons Attribution (CC-BY) license (http://creativecommons.org/licenses/by/4.0/).
